# An Elderly Case of Altered Metabolic Profile Presenting With Respiratory Distress: A Radical Display

**DOI:** 10.7759/cureus.46818

**Published:** 2023-10-10

**Authors:** Ankita Thakur, Sanket S Bakshi, Swaroopa Chakole

**Affiliations:** 1 Department of Medicine, Jawaharlal Nehru Medical College, Datta Meghe Institute of Higher Education and Research, Wardha, IND; 2 Department of Community Medicine, Jawaharlal Nehru Medical College, Datta Meghe Institute of Higher Education and Research, Wardha, IND

**Keywords:** arterial blood gas, perfusion, alveolar injury, metabolic acidosis, respiratory distress

## Abstract

Acute respiratory distress syndrome (ARDS) is a pulmonary pathology that itself can harm and further lead to many other significant hazardous sequelae. Pulmonary vasculature can be distressed by several diseases, but among all the causes, sepsis is one of the main culprits. Its consequences include significant alveolar injury, refractory hypoxemia, ventilation-perfusion mismatch, and destruction of the alveolar-capillary membrane. Dyspnea with diffuse infiltration on a chest X-ray is the most prevalent clinical symptom. Here, we discuss a case of a 62-year-old male patient who presents with ARDS and metabolic anomalies. The patient was treated medically with drug regimens.

## Introduction

Acute respiratory distress syndrome (ARDS) is a clinical condition that comprises widespread lung discomfort, hypoxemia, and severe dyspnea that develops rapidly. Pulmonary infiltrations, contusions, and abnormal radiological findings identify this condition [1]. Up to 60 people per 100,000 experience ARDS annually [2]. Patients with ARDS account for 10% or less of all admissions to critical care units. Patients may have a much higher risk of developing this prognosis if they have had previous chronic medical conditions or pathologies connected to their surgical procedures. In severe cases of ARDS, about 40% of the patients have a better prognosis [1,3]. The PaO2/FiO2 ratio, which compares the partial pressure of oxygen to the proportion of inspired oxygen, is used to determine the severity of ARDS. The average PaO2/FiO2 ratio is 500 [3]. A higher ratio indicates better respiratory exchange. The mainstay of the treatment plan consists of many therapeutic modalities, including treatments to increase pulmonary compliance.

## Case presentation

**Figure 1:**
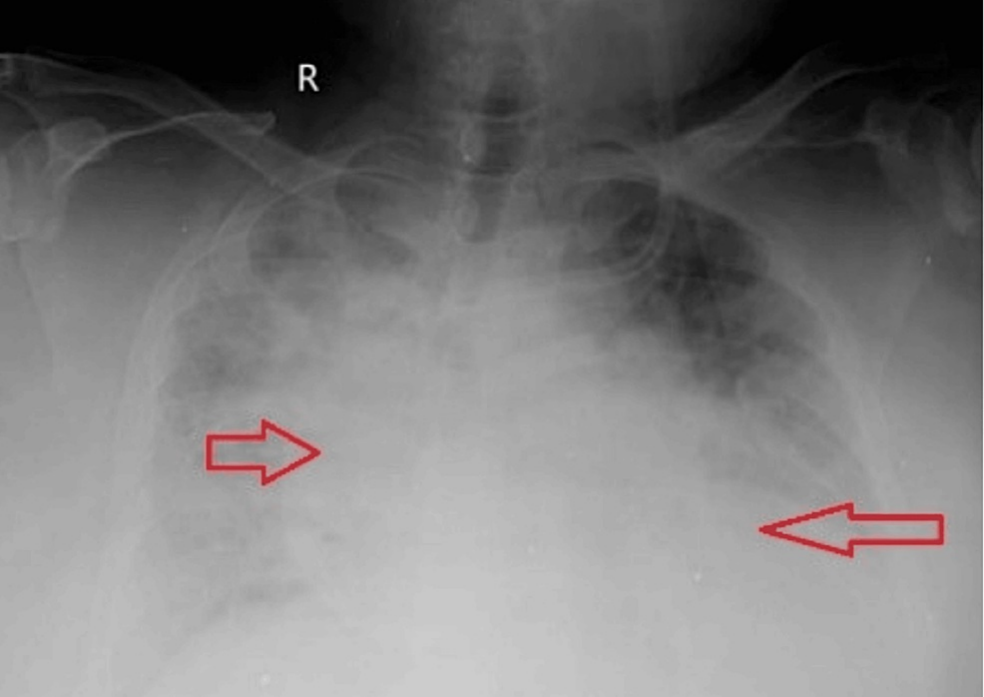
Diffuse bilateral patchy opacities seen on PA view of chest X-ray PA, poster anterior

A 62-year-old male patient was reported to our emergency department with complaints of dyspnea at rest and altered sensorium. The patient experienced these complaints for three years, and it progressively worsened to the stage which made him unable to carry out his daily activities. The patient had been suffering through diabetes mellitus and hypotension. He has been suffering through complaints of cough for around a couple of months, which was dry in nature, with no seasonal or diurnal variations. The systemic examination revealed an elliptical chest, bilateral equal chest rise, resonant notes, no voice resonance, and atypical sounds. A chest X-ray was advised, which revealed diffuse bilateral patchy opacities without cardiomegaly, as shown in Figure 1. 

Additional investigation revealed that the patient was in uncompensated respiratory acidosis, and due to a potential respiratory collapse, the patient had to be intubated immediately upon presenting to the emergency department and transferred to the intensive care unit (ICU). Upon initial blood tests, the serum parameters were seen as deranged, as shown in Table 1.

**Table 1:** Deranged serum parameters

Serum parameter	Value	Reference range
Blood pH	7.2	7.35-7.45
Partial pressure of CO2 (PaCO2)	55 mmHg	38-42 mmHg
D-dimer	690 ng/mL	220-500 ng/dL
Serum urea	48 mg/dL	5-20 mg/dL
Creatinine	2.7 mg/dL	0.7-1.3 mg/dL

Before the management protocol was initiated, the lab investigations were done, which are depicted in Table 2.

**Table 2:** Laboratory investigations

Serum parameter	Levels recorded	Normal range
White blood cell count	12.4 x 10^5^/mL	4.0-11.0 x 10^5^/mL
Hemoglobin	10.0 g/dL	12.0-17.0 g/dL
C-reactive protein (CRP)	15 mg/dL	0.3-1.0 mg/dL
Na+	122 mEq/L	135-145 mEq/L
K+	4.9 mmol/L	3.5-5.5 mmol/L
Lactate	1.3 mmol/L	0.5-2.0 mmol/L
Platelets	1.6 x 10^5/^µL	1.5-3.0 x 10^5^/µL

Bi-level positive air pressure was used to aid mechanical ventilation and 15 L/min of oxygen. Alveolar infiltrates and diffuse, patchy opacities on bilateral chest X-rays worsened. Despite not being medicated, the patient was drowsy and unresponsive to verbal stimulation while in the ICU. On the third day after intubation, he was using the ventilator at its assist control mode with a tidal volume of 6 mL/kg ideal body weight, a respiratory rate of 25/min, a flow rate of 60 L/min, positive end-expiratory pressure of 18 cm H2O, and 70% fraction of inspired oxygen (FIO2). He also required more oxygen. The patient initially received a loading dose of furosemide, a 40 mg injection stat. The palliative care team explained to the patient’s family the medical futility of continuing to treat the patient, given his advanced age and numerous comorbidities (the patient was unlikely to ever wean off from external respiratory support and was unresponsive despite lack of sedation), and the family gave their consent for the cessation of care. The patient was given repeated doses of midazolam (5 mg total IV push) and morphine (10 mg total IV push) a week after being admitted to the hospital. He died quietly once the external respiratory support was removed.

## Discussion

The recent decrease in the prevalence of ARDS death rates is mostly attributable to significant advancements in the care of critically ill patients [4,5]. The medical advancements in drug regimens and the much improved diagnostic procedures have been the reasons behind the lowered incidence of pulmonary pathologies. This makes it vital to pay particular attention to the detection and treatment of underlying diseases, the elimination of pointless procedures and complications, and the adoption of standardized bundles of care for ICU patients while providing care for these patients [6,7]. Sepsis, being one of the foremost predisposing factors to ARDS, was not among the triggers for the pathology, in our case, and is one of the foremost reasons for presenting it. The alveolar-capillary membrane is created by the union of the alveolar epithelial lining and the microvascular endothelium. Damage to the type I alveoli cells and the microvascular endothelium results in increased capillary permeability and an influx of protein-rich fluid into the alveolar space, which further induces interstitial and alveolar edema [8]. Less surfactant is generated as a result of the type II alveolar cells being damaged. Surfactant has a significant role in maintaining the lung’s compliance [9,10]. Therefore, a decrease in the production of surfactants may result in a decrease in lung compliance and alveolar collapse [11]. This further results in lowered ventilation due to the reduction in the alveolar capacity, resulting in a wide array of symptoms.

Additionally, it exacerbates hypoxia and causes an imbalance between breathing and perfusion. The fibro-proliferative stage is the third stage. In the lung, collagen is deposited and cells granulate [12]. Pulmonary capillaries are damaged and destroyed, and alveoli enlarge and take on an uneven shape. The final step is the remodeling or resolution phase. Type II alveolar cells expand and create surfactant when intra-alveolar fluid travels from the alveolus into the interstitial [13,14]. Utilizing medical care is advised. Finding and treating the underlying cause, such as sepsis brought on by intravenous lines, urinary catheters, abscess drainage, and necrotic tissue debridement, constitutes the primary medical therapy [15,16]. Deep vein thrombosis (DVT), hospital-acquired infections, and pressure ulcers should all be avoided. The second essential ARDS treatment plan is mechanical ventilation. Numerous studies demonstrate that patient mortality is reduced by protective lung breathing practices.

## Conclusions

A diverse array of exogenous, along with endogenous factors, play the role of triggers that escalate the pathological cascade, deteriorating the prognosis of ARDS. The pathophysiology of this condition primarily consists of the body’s reaction to irritants that are present and have an impact on the pulmonary structure and membranes, leading to an abnormal radiological image. The management protocol includes several therapeutic regimens, such as fluid control, mechanical ventilation, and glucocorticoids, to treat the immune hyperreaction.
